# Neurovascular Signals Suggest a Propagation Mechanism for Endogenous Stem Cell Activation Along Blood Vessels

**DOI:** 10.2174/1871527311201070805

**Published:** 2012-11

**Authors:** Jimmy Masjkur, Maria Adele Rueger, Stefan R Bornstein, Ron McKay, Andreas Androutsellis-Theotokis

**Affiliations:** 1 Department of Medicine, University of Dresden, Dresden, Germany; 2 Department of Neurology, University of Cologne, Germany; 3 Lieber Institute for Brain Development, Baltimore, USA; 4 Center for Regenerative Therapies Dresden, Dresden, Germany

**Keywords:** Endogenous, neural stem cells, brain, degenerative disease.

## Abstract

Stem cell – based therapies for central nervous system disorders are intensely pursued. Such approaches can be divided into two categories: Transplantation-based, and those that aim to pharmacologically target the endogenous stem cell population in the tissue. Endogenous stem cell – based strategies avoid the problem of immune incompatibility between the host and the grafted cells. They also avoid the placement of a large amount of cells in confined areas, a manipulation which alters the characteristics of the neurovascular microenvironment. We show here that massive pharmacological activation (increase in cell numbers) of the endogenous neural stem cell population in the adult rodent brain maintains the cytoarchitecture of the neurovascular niche. Distances between adjacent stem cells (identified by expression of Hes3) are maintained above a minimum. Hes3+ cells maintain their physical association with blood vessels. These results also suggest a mechanism by which the activation signal from the lateral ventricle can be propagated to areas a long distance away from the lateral ventricles, through autocrine/paracrine actions between adjacent Hes3+ cells, along blood vessels. Finally, powerful effects of angiopoietin 2 on Hes3+ cells help explain the prevalence of proliferating endogenous neural stem cells close to the subventricular zone (an area of high angiopoietin 2 concentration) and the quiescent state of stem cells away from the ventricles and their tight physical association with blood vessels (which express high levels of angiopoietin 1, a cytokine that opposes angiopoietin 2 functions).

## INTRODUCTION

The adult mammalian central nervous system retains a population of immature cells (multipotent stem cells and more restricted neural progenitor cells). The exact numbers and location of these cells are debated but recent reports suggest that these cells exhibit a widespread distribution and are highly relevant to the normal and diseased state of the brain and spinal cord. These findings are generating excitement as they suggest newly found, innate potential of neural tissues to repair themselves when challenged by injury and disease. Understanding the mechanisms that regulate these cells will provide means to manipulate them in the hope of improving functional outcome in neurological disorder patients.

It is commonly agreed that in the adult mammalian brain, the subventricular zone (the area lining the lateral ventricles of the brain) is home for multipotent neural stem cells; stem cells or progenitor cells are also found in the dentate gyrus of the adult hippocampus [1-2]. Both these areas are neurogenic and provide new neurons involved in olfaction and aspects of learning/memory throughout life. Perhaps because these areas contain actively dividing and differentiating cells, they were the first to be identified as stem cell niches. An experimental advantage with these areas was the ability to use proliferation markers, an approach which identified the generation of new neurons in the adult brain.

Neurogenesis and stem cell numbers can be manipulated by insults including disease, ischemia, and seizures, as well as by exogenous factors [15, 19, 22, 43-45]. The readiness of these cells to respond to so many stimuli suggests that their roles are of consequence. To understand how to manipulate these cells by exogenous means, several labs take clues from the microenvironment in which these cells reside [6, 8, 38, 39, 46-61]. Endogenous neural stem cells associate with blood vessels; this observation suggested, and subsequent work confirmed that vascular signals control the numbers and functions of stem cells.

In addition to using clues from the microenvironment, aspects of their signal transduction requirements can be elucidated by considering how the dual and characteristic property of stem cells (self-renewal and multi-potential) can be encoded within the known framework of signaling pathways. For example, we previously showed that neural stem cells exhibit unique signaling requirements relating to the signaling molecule / transcription factor STAT3 [38]. Specifically, many cell types in the body heavily rely on STAT3 phosphorylation on the tyrosine residue for survival; in contrast, STAT3 phosphorylation on the serine residue is dispensable [62]. However, in neural stem cells, STAT3-Tyr posphorylation induces differentiation, suggesting that these cells rely only on the serine phosphorylation site for STAT3 – mediated survival [63-65]. These initial results led to the elucidation of non-canonical signaling pathways that are of great importance to the survival and numbers of neural stem cells but of lesser importance to other cell types [38, 39, 47, 60, 61].

Here we present data supporting a hypothesis that signals from the microenvironment of endogenous neural stem cells and a signal transduction pathway downstream of these signals is a determinant of the location of these cells. The same signals and pathways also provide a mechanism to propagate the activation signal from a starting location to vast distances from it. Importantly, pharmacological manipulations can stimulate this mechanism, providing a powerful and widespread mechanism with therapeutic relevance.

The complex and reciprocal interactions among different cell types within the neurovascular niche suggest that a sensitive balance of neurovascular signals determines the state of the stem cell within the niche (e.g., quiescence, proliferation, differentiation) [66]. In fact, treatments with vascular signals including the Notch receptor ligand Delta4 and the Tie2 receptor ligand angiopoietin 2 induce vast increases in the number of stem cells *in vitro* and *in vivo* [60]. Despite these powerful effects, the cytoarchitecture of the neurovascular niche is not disrupted as tight physical associations between stem cells and blood vessels are maintained. As we report here, the density of neural stem cells along blood vessels is maintained within a particular range. Such limits may be missing from certain transplantation approaches where large numbers of cells are grafted into small volumes of tissue, disrupting the microenvironment and the inherent mechanisms that help avoid excessive stem cell proliferation; one such mechanism, as we have suggested in the past, is the pericyte – derived Tie2 receptor ligand, angiopoietin 1.

The angiopoietin system is a major determinant of angiogenesis [67, 68]. Angiopoietin 2 (Ang2) is produced by the vascular endothelial cells and has a pro-angiogenic function. Angiopoietin 1 (Ang1) is produced by pericytes that cover blood vessels and opposes Ang2 function. This may be an elegant system that promotes angiogenesis when blood vessels are immature (i.e. not yet covered by pericytes, for example, following injury and subsequent angiogenesis), and which halts angiogenesis when blood vessels are matured (and covered with pericytes). Adding to the elegance of this mechanism, endogenous neural stem cells also respond to Tie2 stimulation by Ang2 [39], and this may synchronize the activation of angiogenesis with that of neural stem cells to maximize the efficiency of the regenerative response in the adult neural tissue.

## BACKGROUND - A SIGNALING LOGIC

### Vascular Signals that Regulate Endogenous Neural Stem Cells

In the central nervous system, neural stem cells are part of the neurovascular niche [66]. This is a microenvironment that consists of blood vessels and neural cells; several cell types are involved and many of these can affect others through secreted and membrane-bound factors. These complex inter-cellular interactions (some diffusible and some membrane-bound) may be determinants of many aspects of the niche, including the density of particular cell types, the proximity of different cell populations, and their readiness to respond to stimuli (e.g. injury and pharmacological manipulations).

The involvement of blood vessels in the regulation of stem cells is a phenomenon that extends beyond the central nervous system. For example, germ cell layer stem cells also associate closely with blood vessels [69] and hematopoietic stem cells are reported to receive direct signals from vascular endothelial cells in the bone marrow [70]. Intriguingly, some of these signals may be common to those that also regulate neural stem cells in the adult brain, including Notch and Tie2 receptor ligands [38, 39, 60]. These results suggest that common signaling mechanisms may regulate immature cells in different tissues.

Endogenous neural stem cells in the adult mammalian brain express receptors for a multitude of factors. These factors are derived from several different cell types in the neurovascular niche, suggesting that neural stem cells can respond to several types of change in the tissue. Fig. (**1**) provides a schematic diagram with several interactions among neural stem cells and other cells in the neurovascular niche. The diagram shows a neural stem cell (middle of diagram, large cell) with several receptors on the plasma membrane and key intracellular signaling molecules. In green are elements of the signaling pathway that lead to an increase in the numbers of stem cells; in red are pathways that oppose stem cell number expansion. Light green contoured signs represent pharmacological treatments that increase neural stem cell numbers [38, 39, 47, 60, 61, 71].

*In vitro* and *in vivo* work has established that Notch signaling is a powerful mediator of survival and cell number increases for neural stem cells. In cultured neural stem cells from the fetal and adult rodent brain, treatment with soluble forms of Notch receptor ligands (Delta4, Jagged1) promotes cell survival [38]. In the brain, a major source of Delta4 is the vascular endothelial cell [72]. Because native Notch receptors and their ligands are plasma membrane – bound, close proximity between cells is required for Notch signaling activation. It is possible that endothelial Delta4 and Notch receptors on neural stem cells play a role in the physical proximity between blood vessels and neural stem cells. For example, they may be part of a homing mechanism that attracts stem cells close to blood vessels. A homing hypothesis is supported by studies showing that irradiation of the adult mammalian brain disrupts the physical association between blood vessels and neural progenitor cells [73].

Vascular endothelial cells are also sources of the pro-angiogenic cytokine angiopoietin 2 (Ang2) [68]. Ang2 is a secreted cytokine that interferes with the activation of the Tie2 receptor. Work with cultured endothelial cells has shown that Tie2 can be activated (phosphorylated) by the related cytokine Angiopoietin 1 (Ang1) [68, 74]. Ang1 is secreted by pericyte cells which are located around vascular endothelial cells and are part of the blood vessel structures. Ang1 acting on vascular endothelial cells activates endothelial Tie2 receptors and this inhibits angiogenesis. In contrast, Ang2 competes with Ang1 for Tie2 binding sites without being able to activate the Tie2 receptor. As a result, Ang1 can be considered anti-angiogenic and Ang2 pro-angiogenic [67, 68].

In other cell types such as embryonic fibroblasts, both Ang1 and Ang2 can induce Tie2 phosphorylation [68]. We have previously shown that neural stem cells express the Tie2 receptor and that treatment of cultured neural stem cells with Ang2 induces Tie2 phosphorylation and subsequent increase in cell number. Similar results were also obtained *in vivo* [39, 60]. These results suggest that endothelial cells and pericytes, *via *the angiopoietins, may also regulate the numbers and the activation potential of neural stem cells in the tissue.

Blood vessels may operate an additional mechanism (in addition to Notch and Tie2 ligands) by which they affect neural stem cell numbers and activation. Using a novel marker of neural stem cells, the transcription factor Hairy and Enhancer of Split 3 (Hes3 – discussed later), we showed that Hes3+ cells are tightly associated with the vasculature and express high amounts of the insulin receptor [39]. It is possible therefore, that insulin from the circulation may be able to affect neural stem cells in the brain. In support of this, injections of insulin in the lateral ventricles of adult rats induced powerful expansion of Hes3+ cells within days [47].

Additional vascular signals regulate neural stem cells, including the Vascular Endothelial Growth Factor (VEGF) [66]. Intriguingly, vascular-derived signals that increase neural stem cell numbers can be either pro- or anti-angiogenic. As a result, it is possible to independently regulate stem cell numbers and vascular density [39].

### Non-Canonical Signaling Pathways Regulating Neural Stem Cells

In cultured neural stem cells, the pro-expansion signals described above (Delta4, Jagged1, Ang2, and insulin) activate a convergent signal transduction pathway. Canonical Notch signaling involves the proteolytic cleavage of the intracellular domain of the Notch receptor, its release in the cytoplasm, interaction with other proteins and their nuclear translocation to affect transcription of target genes [75]. In cultured neural stem cells, a non-canonical signaling pathway can be activated by soluble forms of Delta4 and Jagged1 [38]. This leads to the rapid and PI3Kinase – dependent phosphorylation of Akt, within 3 minutes. Subsequently, mTOR is phosphorylated (within approximately 20min), followed by STAT3 on the serine residue. Insulin activates the same pathway as it also induces rapid phosphorylation of Akt and subsequent targets. Ang2 also leads to STAT3-serine phosphorylation but through a different input: Ang2 reduces phosphorylation of the p38 MAP kinase which opposes STAT3-serine phosphorylation [39, 60]. Overall, non-canonical Notch signaling, insulin, and Ang2 lead to the phosphorylation of STAT3 on the serine residue. Critically, this happens in the absence of any phosphorylation on the tyrosine residue of STAT3, as this is known to induce the differentiation of neural stem cells to the astroglial fate, by inducing the expression of glial-specific genes, including Glial Fibrillary Acidic protein (GFAP) [63-65]. STAT3-serine phosphorylation in the absence of STAT3-tyrosine phosphorylation, therefore, induces the expansion of neural stem cell numbers while protecting their self-renewal state.

### STAT3: Regulator of Self-Renewal and Differentiation Through Different Phosphorylation Sites

The importance of STAT3-serine phosphorylation can be shown by transient genetic manipulation of cultured neural stem cells using STAT3 constructs [38]. STAT3 constructs that cannot be tyrosine-phosphorylated have no effect on their survival, as expected. In contrast, constructs that cannot be serine-phosphorylated cause cell death. In accordance, when the serine at the phosphorylation site is mutated to a glutamate (to mimic a constitutively phosphorylated serine residue), cell number significantly increases. These results show that two phosphorylation sites on the same molecule, 22 amino acid positions apart, mediate different functions that are critical to the self renewal, survival, and differentiation potential of neural stem cells.

We have used phosphorylation of STAT3 on the serine residue in the absence of phosphorylation on the tyrosine residue as a predictor for additional treatments that increase neural stem cell numbers. JAK kinase is the direct tyrosine kinase that phosphorylates STAT3 on the tyrosine [62]. JAK kinase activation also leads to p38MAP kinase phosphorylation which, as described earlier, opposes STAT3-serine phosphorylation in cultured neural stem cells [38]. A JAK kinase inhibitor, therefore, would be expected to protect STAT3 from tyrosine phosphorylation and inhibit p38MAP kinase from opposing STAT3-serine phosphorylat-ion. Indeed, JAK kinase inhibition was established as a mediator of cultured neural stem cell survival [38]. As expected, a p38 MAP kinase inhibitor also increases the numbers of cultured neural stem cells.

### Hes3: An Indirect Target of Non-Canonical Notch Signaling

Hes1 and Hes5, two members of the Hes/Hey gene family of basic helix-loop-helix transcription factors are direct targets of the cleaved intracellular domain of Notch [76-78]. Hes3, another member of the family has been established as a non-direct target of downstream of Notch activation. We have previously shown that treatments that induce STAT3-serine phosphorylation induce transcription of Hes3 within one hour [38]. The functions of Hes3 are largely unknown. Hes3 null mice develop normally with very mild side effects [77]. When Hes3 is deleted in mice already lacking Hes1 and Hes5, then precarious differentiation of neural precursors is observed in the developing embryo, suggesting a role of Hes3 in the maintenance of the stem cell / precursor cell population in the brain [76]. At the level of whole mount in situ detection, Hes3 was shown to be lost at around the mid-gestation period in mouse embryos [77]. In the adult brain, we showed that the subventricular zone of mice and rats is enriched, relative to the levels of the average brain, in Hes3 protein and mRNA [38]. This result established that Hes3 expression is maintained, even in the adult, although at reduced levels, relative to the developing organism. Immunohistochemical analyses of adult rodent and primate brains confirmed this result. Hes3+ cells were detected throughout the adult brain and spinal cord, with a greater incidence in the subventricular zone [39]. These results suggested that Hes3 identifies the endogenous neural stem cell population which is most prominently, but not exclusively, found in the subventricular zone. In accordance with other reports, Hes3 confirmed the subventricular zone as the richest area in endogenous neural stem cells in the adult mammalian brain [38, 39]. Hes3 immunodetection, however, allows the identification of cells outside the subventricular zone, in areas of both grey and white matter. The cytoarchitecture of Hes3 cells is similar in all areas and these cells exhibit a very tight physical association with the vasculature, suggesting similar properties among Hes3+ cells in these regions. Indeed, when adult brain and spinal cord tissue was placed in culture following microdissection of particular areas, Hes3+ cells were able to grow; these cells co-expressed other stem cell / precursor markers including Sox2 and nestin and, under clonal conditions, differentiation induced by withdrawal of mitogen generated all three main cell types of the neural tissue: neurons, astrocytes, and oligodendrocytes. Differentiation also induced the loss of Hes3 expression, establishing Hes3 as a novel marker of multipotent neural precursors.

These results do not preclude that all Hes3+ cells are identical in all respects. For example, the morphology of neurons generated from Hes3+ cells obtained from the adult rat spinal cord is different than that from the subventricular zone. Spinal cord – derived neurons exhibit morphologies reminiscent of motoneurons and interneurons, and express LIM homeobox genes (islet1, islet2) [60].

When fetal mouse neural stem cells in culture are transfected with Hes3 constructs, an elevation in sonic hedgehog (shh) protein is observed within two days [38]. This result is in accordance with a role of Hes3 in the self-renewal of neural stem cells as shh has mitogenic properties [79]. This result also suggests a role of Hes3 in the trophic support of neurons, as shh has neuroprotective roles for neurons, including injured dopamine neurons in models of Parkinson’s disease [80]. It will be of interest to study other target genes downstream of Hes3, in order to assess additional mechanisms by which Hes3+ cells may be influenced by their microenvironment and may, in turn, protect injured neurons.

### Neurons and Astrocytes may Also Regulate Endogenous Neural Stem Cells Through Signaling Mechanisms Involving STAT3

Cultured neural stem cells from the adult central nervous system can be readily manipulated by treatment with exogenous basic Fibroblast Growth factor (bFGF) and Ciliary Neurotrophic Factor (CNTF) [65]. In fact, bFGF is a mitogen for these cells and withdrawal of bFGF induces their differentiation; CNTF is a potent inducer of their differentiation to the astroglial fate, *via *phosphorylation of STAT3-tyrosine. Within the neurovascular niche, both neurons and astrocytes produce bFGF. Astrocytes, in addition, also produce CNTF. The endogenous neural stem cell, therefore, finds itself in a sensitive position where imbalances in the signals that it receives from nearby cells determine whether it will retain its ability to self-renew or it will be induced to differentiate.

### Neural Stem Cell Feedback to Blood Vessels

Interactions between the vasculature and neural stem cells are likely reciprocal, and a signaling mechanism can be pieced together from various observations. In endothelial cells, shh regulates the expression of the angiopoietins [81]. It is possible that shh produced by neural stem cells may influence cells of the vasculature to express the angiopoietins (Ang1 from pericytes and Ang2 from endothelial cells). The angiopoietins, in turn, may regulate the neural stem cells. Depending on which cell type of the vasculature prevails in terms of their activation, a different angiopoietin balance will be set and the outcome on the neural stem cell may vary between increased quiescence and proliferation.

### Cholera Toxin: Powerful Activator of Neural Stem Cell Proliferation

Recently we showed that cholera toxin is a potent activator of neural stem cell proliferation in culture [61]. Cholera toxin consists of two subunits. Subunit B (there are 5 B subunits) binds to GM1+ gangliosides which are concentrated in certain lipid rafts on the plasma membrane. As lipid rafts may internalize as a means of recycling plasma membrane and its components, cholera toxin is found membrane-bound inside the cell. There, the A subunit is released into the cytoplasm where is activates adenylate cyclase and elevates cAMP levels [82-85]. Cholera toxin has several functions in addition to adenylate cyclase activation, including the modulation of intracellular trafficking and activation of the Endoplasmic Reticulum associated degradation (ERAD) pathway [86]. We reported that treatment of neural stem cell cultures with cholera toxin induces the nuclear localization of Hes3 [61]. In addition, the treatment increases cell numbers by many times. In fact, cholera toxin can maintain cultured cells in the proliferative state for at least one week following the withdrawal of the mitogen bFGF, suggesting a powerful role in proliferation.

These findings provide a mechanism by which different cell types within the neurovascular niche mutually interact. Soluble and membrane-bound signals may also regulate the physical proximity of cells to each other. The multitude of receptors on the surface of neural stem cells render these cells malleable to signals from several nearby cell types including vascular endothelial cells, pericytes, neurons, and astrocytes. In addition, insulin receptors on these cells may allow the systemic circulation of the organism to affect their function and numbers, providing a direct means for the metabolic state of the organism to affect endogenous neural stem cells.

In this paper we address a specific feature of the neurovascular niche: Why the majority of endogenous neural stem cells are located in the subventricular zone lining the lateral ventricles of the adult brain. Specifically, we assess how the microenvironment in this area differs from the parenchyma of the brain (e.g., the striatum) and whether aspects of the neurovascular niche described above can help explain this phenomenon. We report that the ratio of Ang1/Ang2 expression differs significantly between the subventricular zone and areas away from the subventricular zone, suggesting a mechanism that explains the non uniform distribution of these cells in the adult central nervous system.

In addition, we extend these observations to suggest a mechanism by which an activation signal can be propagated from Hes3+ cell to adjacent Hes3+ cell along the surface of blood vessels, through a sequential autocrine/paracrine mechanism. Such a mechanism would help explain the rapid and massive activation of endogenous neural stem cells throughout the central nervous system that can be observed following injection of soluble factors (small molecules and/or proteins) into the lateral ventricles of adult mice and rats [38, 39, 47].

## METHODS

### *In Vivo* Manipulations in Adult Rats

These experiments were performed as previously described [39]. Treatments were approved by National Institutes of Health (NIH) guidelines (where they were performed), conforming to the Guide for the care and use of laboratory animals of the U.S. National Institutes of Health (NIH publication 85-23, revised 1996). Male adult (3–6 months) Sprague-Dawley rats (Charles River Laboratories), weighing 250–350 g, were used. Five microliters of different drugs were stereotactically injected into the right lateral ventricle using the following stereotaxic coordinates: Bregma AP −0.9 mm, ML −1.4 mm, VD +3.8 mm. The following reagents were used in combination: Dll4 (2 mg/mL), Ang2 (1 mg/mL), insulin (8 mg/mL), Jak-Inhibitor (20 μM). Animals recovered from the anesthesia and were put back into their home cages, with access to food and water ad libitum. Rats received i.p. injection of the tracer 50 mg/kg BrdU every 12 h for 5 days beginning on day 1 post-op to label dividing cells. In this study, we did not assess BrdU incorporation. Rats were sacrificed 5 days after treatment.

### Neural Stem Cell Culture

E13.5 neural stem cells were grown as previously described [60]. Cells were expanded in serum-free DMEM/F12 medium with N2 supplement and FGF2 (20 ng/ml) for 5 days under 5% oxygen conditions and were re-plated fresh or from frozen stocks at 1,000–10,000 cells per cm^2^. FGF2 was included throughout our experiments, unless otherwise stated. Adult rat (3–6 months old) or adult mouse (2–4 months old) SVZ Neural Stem Cell (NSC) cultures were grown in the same medium as the fetal cultures.

### Immunohistochemistry

Under deep anesthesia, animals were perfused transcardially with a rinse of saline, followed by 4% formaldehyde fixative (pH 7.4). Brains were removed immediately, stored in the fixative solution overnight, and then in 30% sucrose for 3 days. Brains were frozen-sectioned at 12 or 30 micrometers. Immunofluorescence analysis was carried out using standard protocols and Alexa fluor – conjugated secondary antibodies. Images were acquired using a Zeiss Apotome microscope to achieve confocality. The images shown are overlays of optical sections obtained from the same slide.

### Reagents

We used the following reagents and antibodies: FGF2 (233-FB), mouse Dll4 (1389-D4), fibronectin (1030-FN), human angiopoietin-2 (623-AN), from R&D; JAK Inhibitor I (420099), from Calbiochem; Polyornithine (P-3655), insulin (I9278) from Sigma; Alexa-Fluor-conjugated secondary antibodies from Invitrogen; DAPI (D-8417) from Sigma, ECL reagents (34080) from Pierce, polyacrylamide gradient gels from Invitrogen, HRP-conjugated secondary antibodies from Jackson Immunoresearch, and general chemicals from Sigma. For immunohistochemical staining and Western Blotting, we used antibodies against the following markers: Hes3 (25393), Tie2 (sc-324), Tie2 (sc-31266), STAT3 (482), pSer727-STAT3 (8001-R), pTyr705-STAT3 (7993) from Santa Cruz; pTie2 (AF2720) from R&D Systems; α-tubulin, Tie2 (R&D, AF313), pTie2 (R&D, AF2720) from (Sigma, T-6074), p38 (9212) from Cell Signaling.

### Statistical Analysis

Results shown are the mean ± S.D. Asterisks identify experimental groups that were significantly different (p-value<0.05) from control groups by the Student's t-test (Microsoft Excel), where applicable.

## RESULTS

### Ang1 *vs* Ang2: Opposing Effects on Neural Stem Cells

As described above, the angiopoietin system is a regulator of both angiogenesis [67, 68] and endogenous neural stem cell numbers [39]. Ang1, secreted from pericytes, induces the phosphorylation of the Tie2 receptor on vascular endothelial cells, and this stimulus opposes the generation of new blood vessels. In contrast, Ang2, produced by vascular endothelial cells themselves, competitively opposes the action of Ang1 with a net result the induction of angiogenesis. This is a very elegant system that promotes angiogenesis in immature blood vessels (which do not yet have pericytes associated with them) and inhibits angiogenesis in mature blood vessels (which, now, have pericytes secreting Ang1). Our finding that neural stem cells also express the Tie2 receptor suggests a mechanism whereby injured tissue induces simultaneously the generation of new blood vessels and new neural stem cells, as would be expected from a regeneration paradigm.

**Fig. (2):**
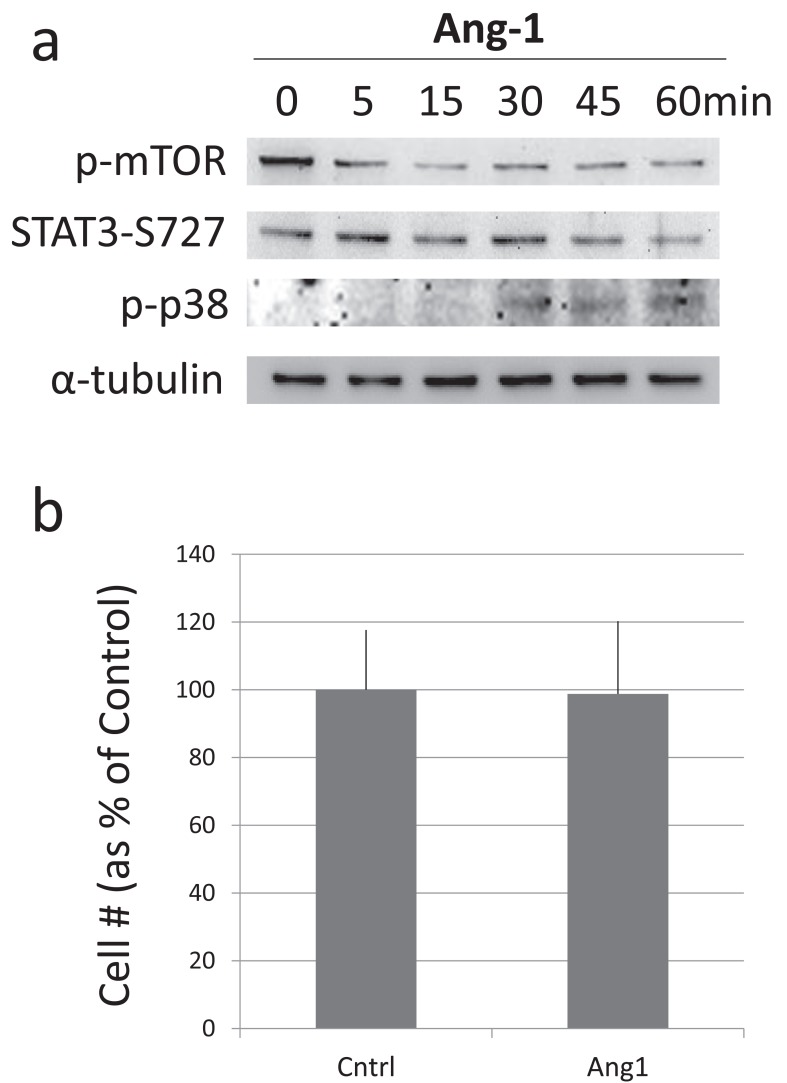
Angiopoietin 1 does not promote the expansion in number of cultured fetal mouse neural stem cells. (**a**) Treatment of cultured neural stem cells with Ang1 results in the time-dependent reduction in the levels of phosphorylated mTOR. STAT3-serine phosphorylation exhibits a slight reduction. p38MAP kinase phosphorylation, in contrast, exhibits a slight increase. (**b**) Treatment of cultured neural stem cells with Ang1 for 5 days has no effect on cell number.

When cultured neural stem cells are treated with Ang2, the Tie2 receptor is phosphorylated [39, 60], similar to the action of Ang2 on embryonic fibroblasts, and unlike its action on human vascular endothelial cells [68]. Following this modification on Tie2, STAT3 is phosphorylated on the serine residue, without any detectable phosphorylation on the tyrosine residue. As expected, cell numbers increase. In contrast to Ang2, treatment with Ang1 does not lead to the phosphorylation of STAT3-serine; in fact, a slight reduction in STAT3-serine phosphorylation is observed (Fig. **2a**). This reduction may be due to a mild increase in p38 MAP kinase phosphorylation which is also observed with similar kinetics (Fig. **2a**). This is because p38 MAP kinase activity has been shown to promote STAT3-serine phosphorylation [87, 88]. As would be expected, treatment of mouse fetal neural stem cell cultures with Ang1 does not change cell numbers (Fig. **2b**). These results show that Ang2 induces the expansion of the endogenous neural stem cell population whereas Ang1 does not have this effect. These observations suggest a mechanism to stimulate the expansion of neural stem cells in the regenerative, angiogenic, phase following injury, and to induce their quiescence in order to avoid uncontrolled growth after the angiogenic phase is completed.

### Ang1/Ang2 Ratios Vary Between the Adult Subventricular Zone and the Brain Parenchyma

Our *in vitro* results showed that Ang1 has no effect on neural stem cell numbers whereas Ang2 strongly increases cell numbers. The effect of Ang2 was extended *in vivo* showing powerful expansion of the endogenous neural stem cell population [39, 60]. These observations suggest that Ang1/Ang2 ratios may be a determinant of the suitability of particular brain areas to home neural stem cells.

The cerebrospinal fluid inside the ventricles of the brain is rich in Ang2 produced by the choroid plexus. We hypothesized that brain areas close to the ventricular wall (e.g. the subventricular zone) may exhibit a higher ratio of Ang2 over Ang1 expression than areas away from the ventricle. To address this, we acquired series of images in the subvenricular zone and in the striatum, immunolabeled for Ang1 and Ang2. Images were acquired during the same preparation and with identical acquisition settings to allow direct comparison of the results, and they were counter-stained for Hes3 to show the location of endogenous neural stem cells. Signal from the Ang1 and Ang2 channels were quantified using imaging software (Photoshop histograms). Whereas these results do not address if there is more Ang1 or Ang2 in absolute values, they do provide a measure of Ang1/Ang2 ratios in different brain areas. We note that images from different areas of the brain (e.g., subventricular zone *vs* stratum) were obtained from the same brain section to control for variations in the levels of immunonofluorescence signal from preparation to preparation. Fig. (**3**) shows examples of the staining described above. An obvious difference is that the levels of Ang2 are much higher at the subventricular zone than in the striatum of the same brain sections, whereas the levels of Ang1 increase. Fig. (**4a**) shows a blood vessel (stained for RECA1) covered with pericytes (stained for Ang1) and Hes3+ cells. Quantifications of Ang2/Ang1 ratios in the subventricular zone, substantia nigra, and striatum are given in Fig. (**4b**). These results correlate a high Ang2/Ang1 ratio to a high incidence of Hes3+ cells in the adult brain.

### Activated Endogenous Neural Stem Cells Maintain a Distance from Each Other

Various pharmacological treatments can increase the numbers of Hes3+ cells in the adult rat brain [38, 39, 47]. These treatments can be administered as single injections in the lateral ventricle and they include Delta4, Ang2, insulin, and a combined treatment that contains Delta4, Ang2, insulin, and a JAK kinase inhibitor. Of these treatments, Delta4 is anti-angiogenic, whereas Ang2 and insulin are pro-angiogenic. In contrast to these single factor treatments, the combined treatment has minimal effects on the vasculature. Here we used the combined treatment to assess the average distance between adjacent Hes3+ cells along the same blood vessel. Examples of images of the activated brain are given in Fig. (**5a, b**>). Using image acquisition software (Zeiss Axiovision) we measured the distances between adjacent Hes3+ cells. Specifically, we measured the distance between the following two points: (a) where one Hes3+ cell comes to contact with the vasculature and (b) where the adjacent Hes3+ cell, which is in contact with the same blood vessel, comes into contact with that blood vessel. The average distance was 28.2 micrometers with a standard deviation of 10.3 micrometers. Rarely (less than 4% of the occasions) were two adjacent Hes3+ cells closer than 10 micrometers from each other. We do not provide measurements of control (saline-injected) brains, as in these cases, Hes3+ cells are fewer by several fold, as previously reported [39]. Our results do not exclude the possibility that adjacent Hes3+ cells may be in physical contact which cannot be easily observed using the immunohistochemical approach used here. This would be an intriguing possibility as it would suggest additional regulators of the distance between adjacent hes3+ cells, possibly including the Delta/Jagged plasma membrane ligand family and their Notch receptors [75]. These results demonstrate that massive activation of the endogenous neural stem cell population can be achieved by pharmacological means and that this approach maintains the principles of the cytoarchitecture of the neurovascular niche, avoiding instances of high immature cell density.

## DISCUSSION

Therapeutic approaches that aim to activate endogenous neural stem cells are increasingly being studied. The hope that the innate repair capabilities of the adult brain may be coaxed pharmacologically is supported by several studies demonstrating beneficial effects in animal models of disease. Such studies also uncover new potential functions of endogenous neural stem cells, including the provision of trophic support to compromised neurons.

Pharmacological treatments can increase the numbers of endogenous neural stem cells by many times. Despite these powerful effects, however, the tissue microenvironment is evidently able to limit local increases in stem cell density and to maximize the volume of the central nervous system where stem cell increases can be measured, thus dissipating the outcome in a large volume of tissue. Specifically, we reported here that despite a large increase in neural stem cell number following pharmacological activation, a distance limit of ~28 micrometers (+/- ~10 micrometers) between adjacent cells along the same blood vessel is maintained. Rarely did we observe Hes3+ cells at a distance of less than 10 micrometers from each other. This may be a critical aspect of approaches that target endogenous stem cells as the essentials of the neurovascular niche are maintained. This may be in contrast to some cell transplantation strategies where large numbers of cells may be grafted in small volumes, resulting in the breakdown of the microenviron-ment relations among different cell types described in Fig. (**1**). If these strategies are not implemented with care to avoid large local grafted cell densities, then the grafted cells may not be able to receive signals from vascular endothelial cells, pericytes, neurons, and astrocytes properly, and they may exhibit abnormal growth. This may be a reason behind unfortunate growths following fetal cell grafting leading to local, graft-derived growths [89].

Elucidation of the signals that a neural stem cell may receive within its microenvironment will help explain the phenomenon of the massive and widespread expansion of the endogenous neural stem cell niche demonstrated following injection of soluble factors in the lateral ventricle. Lateral ventricles are considered relatively convenient areas for the implementation of stereotaxic injections due to their large size. Also, ventricles are in contact with several areas of the brain and spinal cord and treatments injected into the ventricles come into contact with a large surface area in the central nervous system. Third, the largest density of endogenous neural stem cells may be located close to the ventricles; intracerebroventricular injections, therefore, are optimal for access to maximal numbers of stem cells.

These facts do not immediately explain why intracerebroventricular injections of soluble factors, including individual proteins, induce massive and widespread expansion of the endogenous neural stem cell populations throughout the brain and spinal cord. However, assessment of the signaling pathways that regulate neural stem cells can provide possible explanations. The cerebrospinal fluid in the ventricles is rich in Ang2, produced by the choroid plexus. This suggests that a determinant of the subventricular zone as a home for neural stem cells is the high ratio of Ang2/Ang1 in the subventricular zome, relative to other areas further away from the ventricles like the striatum and substantia nigra. Our results presented here show clear differences in Ang2/Ang1 ratios in these areas.

**Fig. (6):**
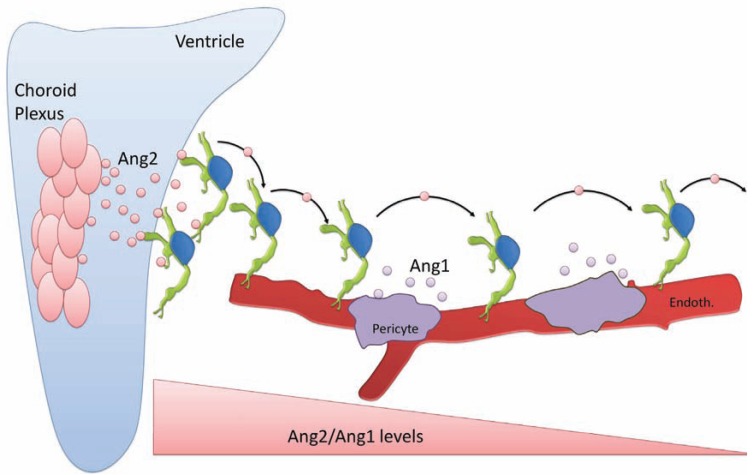
A putative mechanism for the rapid propagation of endogenous stem cell activation throughout the central nervous system, along blood vessels. We propose a hypothetical mechanism, in accordance with the data presented here and in our previous work, to explain how single intracerebroventricular injections of soluble factors (including individual protein species) induce widespread increases in the numbers of Hes3+ cells throughout the adult brain and spinal cord. This model also contributes to the explanation why the incidence of endogenous neural stem cells is higher in the subventricular zones. The cerebrospinal fluid in the ventricles is rich in Ang2 produced by the choroid plexus. As a consequence, the subventricular zone is a brain area with a particular high Ang2/Ang1 ratio. Neural stem cells in the subventricular zone, therefore, exhibit activated signaling pathways that regulate STAT3-serine phosphorylation and, subsequently, Hes3 expression. High Ang2/Ang1 rations may also be a homing signal for neural stem cells which are highly dependent on vascular signals (and may interpret the subventricular zone as a perivascular area, with respect to the signals presented there). Activation of the STAT3- serine/Hes3 pathway (by increased amounts of Ang2, or by another pharmacological stimulation) induces increases in Hes3 expression in endogenous neural stem cells in the vicinity of the stimulation area. Hes3+ cells co-express shh and in cultures of Hes3+ cells, transfection with exogenous Hes3 induces shh increases. In human vascular endothelial cells, shh regulates the expression of the angiopoietins. It is conceivable that Hes3+ cell – derived shh may induce vascular endothelial cells (or even the Hes3+ cells themselves) to produce more Ang2. Given the short distance between adjacent Hes3+ cells along a blood vessel (and their physical association with the blood vessel), a loop involving the angiopoietins and shh may be set and propagated along blood vessels to many areas of the brain.

**Fig. (7):**
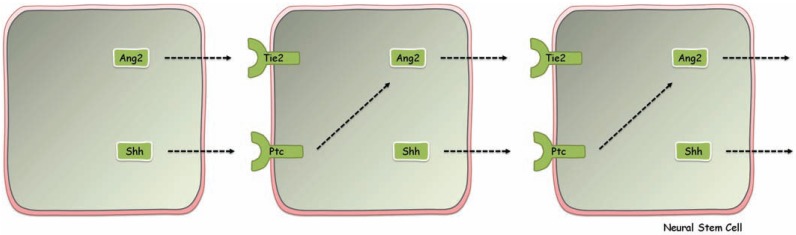
A putative mechanism for the involvement of sonic hedgehog in the propagation of endogenous stem cell activation, along blood vessels. Hes3+ neural stem cells produce Ang2 and due to their close physical proximity in the activated tissue, Ang2 may be able to diffuse from one cell to the adjacent cell on the same blood vessel. This mechanism may be involved in the rapid propagation of the stem cell activation signal. Hes3+ neural stem cells also express the morphogen/mitogen sonic hedgehog. This may be an additional mechanism to ensure powerful and fast signal propagation. In addition, sonic hedgehog is a regulator of the angiopoietins in endothelial cells [81], suggesting the possibility that it may also contribute to increased Ang2 expression in Hes3+ cells, thereby promoting the signal propagation both directly (*via* its receptor Patched, “Ptc”) and indirectly, *via* increased Ang2 release.

Intracellular signaling in neural stem cells can also help explain the widespread effects observed following their pharmacological activation. Cultured neural stem cells express the Tie2 receptor but also Ang1 and Ang2. This suggests that neural stem cells possess an inherent ability to regulate their Tie2 receptor and, consequently, their proliferative state. It is conceivable that Ang2 injected in the ventricle causes local increases in Ang2 in the subventricular zone, beyond a threshold, activation of Tie2 receptors. Tie2 activation leads to increases in Hes3 transcription and, possibly, as in the *in vitro* case, elevated shh production. Shh (or even Ang2 elevated by shh) may then diffuse to the next neural stem cell along the blood vessel with which they associate to continue the cycle to the next cell and propagate the activation signal (Figs. **6**, **7**).

Our results suggest a homing mechanism for endogenous neural stem cells close to the ventricles and a means of propagating an activation signal along blood vessels.

## Figures and Tables

**Fig. (1):**
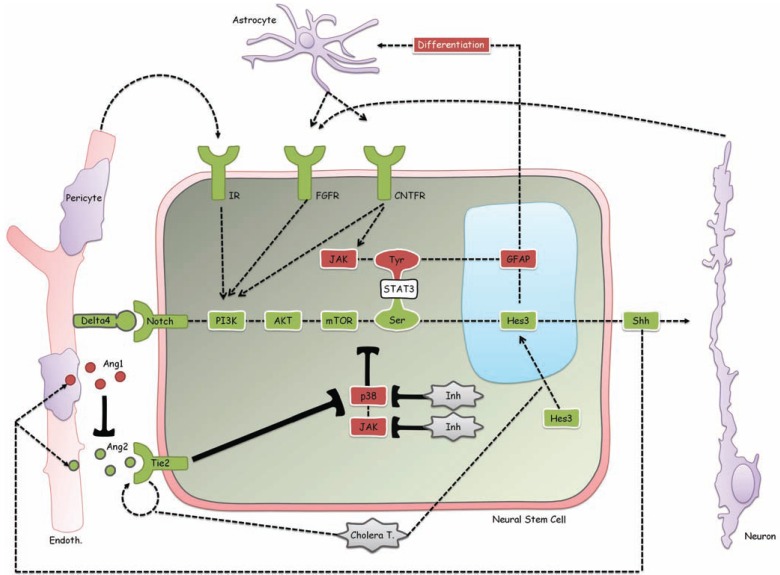
Intercellular interactions within the neurovascular niche. The diagram demonstrates some of the signals derived within the neurovascular niche that regulate cells of the blood vessels (vascular endothelial cells, pericytes), neural stem cells, neurons, and astrocytes. A detailed description is provided in the main text.

**Fig. (3):**
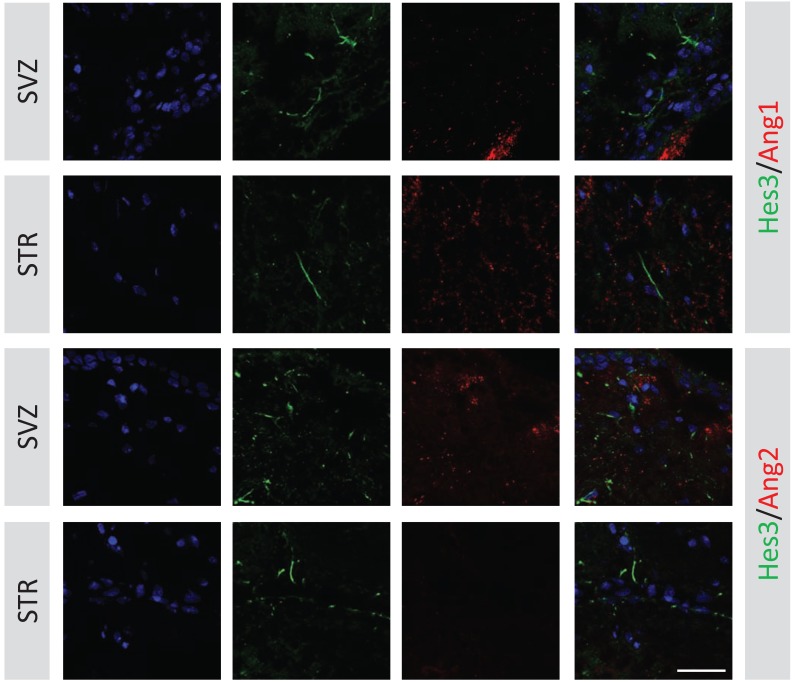
Ang2/Ang1 levels vary in different regions of the adult rat brain. Adult rat brains were stained for Ang1 and Hes3 or Ang2 and Hes3. Different areas of the brain (subventricular zone, striatum) were analyzed using imaging software for the relative levels of Ang1 and Ang2 signal. The results reveal that a higher relative ratio of Ang2/Ang1 characterizes the subventricular zone relative to the striatum. [Blue: DAPI; Error bar: 25 micrometers].

**Fig. (4):**
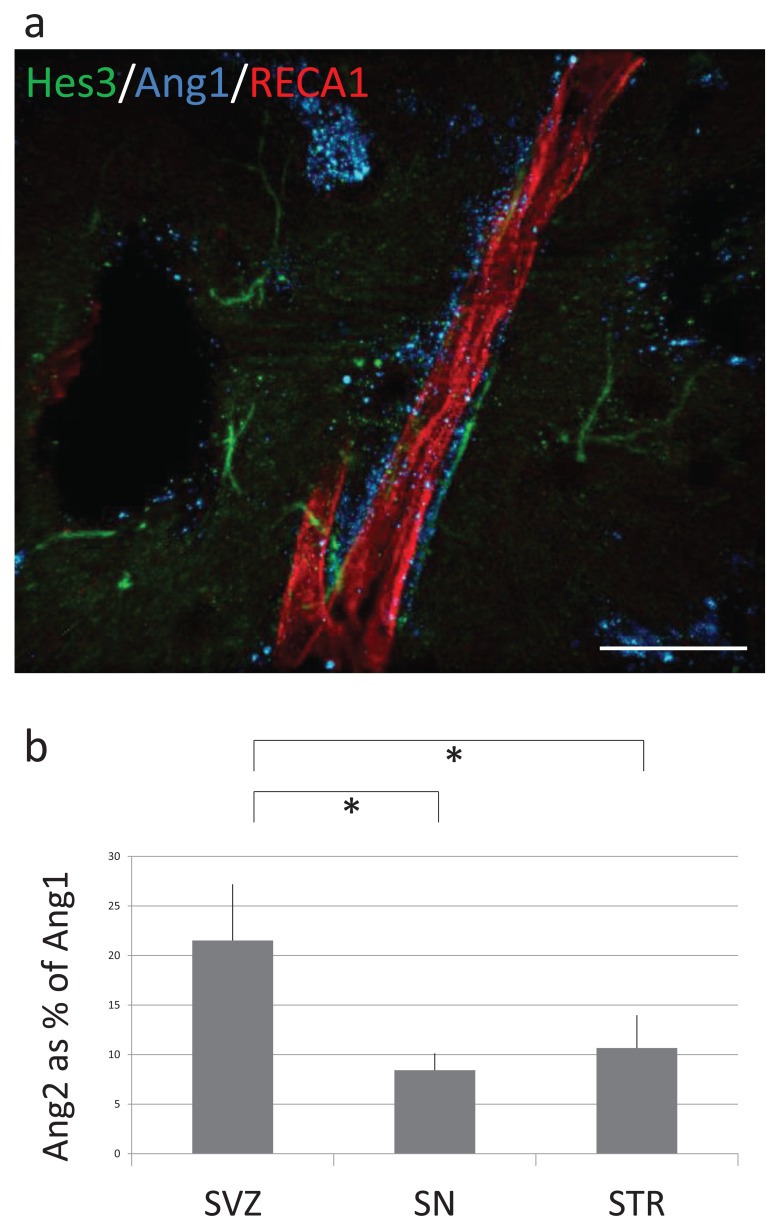
Endogenous neural stem cells in the adult rat brain parenchyma associate with pericyte-covered blood vessels. (**a**) High-power confocal projection image from the adult rat striatum shows a blood vessel (identified by RECA-1 expression) and associated pericytes (identified by Ang1 expression). Hes3+ cells also associate with blood vessels. (**b**) Quantification of the relative Ang2/Ang1 ratio in the subventricular zone, substantia nigra, and striatum shows a significantly higher value for the subventricular zone. [Error bar: 25 micrometers].

**Fig. (5):**
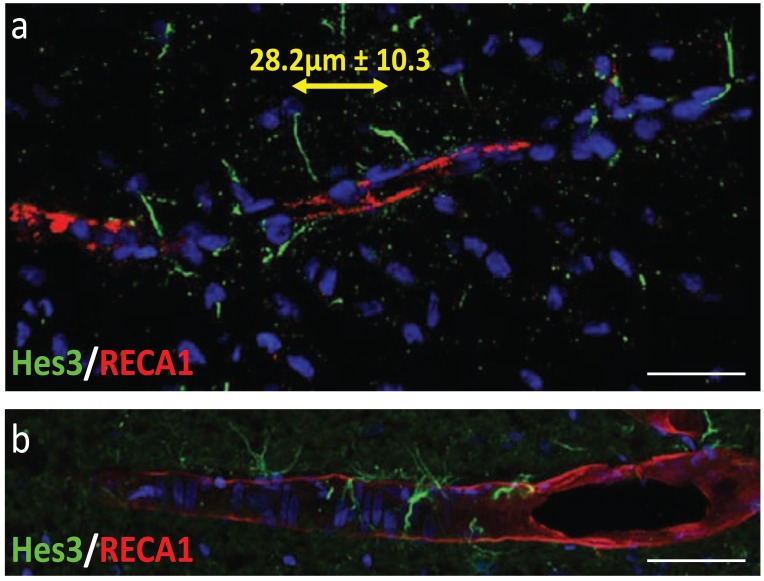
Hes3+ cells associate with the vasculature and maintain ordered distances between adjacent Hes3+ cells. (**a**, **b**) Two examples of Hes3+ cells and their associated blood vessels are shown from the rat brain striatum and substantia nigra, respectively. Five days before tissue collection, rats were administered a single intracerebroventricular dose of the combined treatment (Delta4, Ang2, insulin, JAK kinase inhibitor) to increase the number of Hes3+ cells. These images, therefore, are from the pharmacologically activated adult rat brain. The average distance between adjacent Hes3+ cells on the same blood vessel is 28.2 micrometers with a standard deviation of 10.3 micrometers. Rarely (less that 4% of the cases measured) were two adjacent Hes3+ cells closer to each other than 10 micrometers. [Blue: DAPI; Error bars: 25 micrometers].
